# Growth Performance and Disease Resistance against *Vibrio parahaemolyticus* of Whiteleg Shrimp (*Litopenaeus vannamei*) Fed Essential Oil Blend (Phyto AquaBiotic)

**DOI:** 10.3390/ani13213320

**Published:** 2023-10-25

**Authors:** Tran Thi Tuyet Hoa, Mahougnon Siméon Fagnon, Dang Thuy Mai Thy, Thibaut Chabrillat, Nguyen Bao Trung, Sylvain Kerros

**Affiliations:** 1 Faculty of Aquatic Pathology, College of Aquaculture and Fisheries, Can Tho University, Campus II, 3/2 Street, Can Tho City 90000, Vietnam; dtmthy@ctu.edu.vn (D.T.M.T.);; 2 Phytosynthese, 63200 Mozac, France; thibaut.chabrillat@phytosynthese.fr (T.C.);

**Keywords:** essential oil mixture, AHPND, *Vibrio parahaemolyticus*, *Liptopenaeus vannamei*, growth performance, survival

## Abstract

**Simple Summary:**

Plant-derived essential oils are promising preventive supplements as antibiotic alternatives in aquaculture. In this study, an essential oil blend (Phyto AquaBiotic, abbreviated as PAB) was tested in *Liptopenaeus vannamei* to evaluate its effect on growth improvement and mortality mitigation when infected by *Vibrio parahaemolyticus* (causative agent of Acute Hepatopancreatic Necrosis Disease). Shrimps were fed with two dosages of PAB for 42 days followed by a challenge against a pathogenic strain of *V. parahaemolyticus*. Results showed an improvement in growth performance and reduction in mortality compared to the positive control (unsupplemented with PAB and challenged against *V. parahaemolyticus*). Moreover, the *Vibrio* spp. count in the hepatopancreas of shrimps fed with 2 g/kg of this blend was markedly lower compared to that of the control. To conclude, 2 g/kg PAB contributes in reducing mortality in the context of AHPND outbreaks in whiteleg shrimp (*L. vannamei*).

**Abstract:**

Acute Hepatopancreatic Necrosis Disease (AHPND) is a serious and emerging disease caused by a group of strains of *Vibrio parahaemolyticus* and affects farmed shrimp, particularly whiteleg shrimps (*Liptopenaeus vannamei*). The objective of this study is to assess the effect of dietary supplementation with two dosages of an essential oil mixture (Phyto AquaBiotic, abbreviated as PAB) on growth performance and mortality reduction after challenge against *V. parahaemolyticus*. PAB was mixed with basal diets at rates of 0, 1 and 2 g/kg and fed for 42 days. Each tank was stocked with 100 individuals with experimentation performed in triplicate. The results showed an improvement in growth performance in a dose-dependent manner, specifically regarding daily weight gain, specific growth rate and total biomass, which were significantly improved compared to control (*p* < 0.05). Further, PAB significantly reduced mortalities when challenged against *Vibrio parahaemolyticus* (*p* < 0.05) and decreased *Vibrio* spp. count in the hepatopancreas of infected shrimp. Overall, PAB was efficient in reducing mortalities in cases of disease outbreaks at a rate of 2 g/kg.

## 1. Introduction

Aquaculture, mainly shrimp culture, is among one of the fastest growing sectors in the global food industry [1]. Over the last decades, the industry has experienced significant growth, driven by the increasing demand for seafood and the decline in availability of wild fish stocks. This growth has emerged with many challenges such as sustainability, economic viability and disease outbreaks [2]. From these, disease outbreak is one of the critical challenges and occurs due to the high density of fish/shrimps in a small area. In the shrimp industry, many diseases have emerged since the early years of farming [3]. Viral and bacterial infections are the most devastating disease outbreaks, representing huge losses for the sector. As this sector continues to evolve with intensified systems of production, disease outbreaks remain one of the biggest challenges [4].

Early mortality syndrome (EMS), also called Acute Hepatopancreatic Necrosis Disease (AHPND), is considered as a serious disease in shrimp farming. This disease, caused by *Vibrio parahaemolyticus*, is found in different countries producing *L. vannamei* such as Vietnam, Malaysia, Thailand, Mexico, China and the Philippines [5].

*V. parahaemolyticus* is a Gram-negative bacterial species that causes severe losses in shrimp production [5]. Some external symptoms observed in shrimp infected with AHPND include an empty stomach and a shrunken hepatopancreas (HP). Mortality rate is significantly higher during the first 3 days with disease outbreak occurring within 8 and 45 days after stocking [5]. Currently, antibiotics like tetracyclines, quinolones, trimethoprim, sulfonamides, oxolinic acid and sarafloxacin are used for preventive and/or curative purposes against the bacterial genus *Vibrio* [6]. Obviously, these various antibiotics are commonly known to exhibit some negative impacts on overall health (such as antimicrobial resistance) since some are applied abusively or without following veterinarian recommendation. Presently, a global awareness has started to progressively emerge which has pressured some countries to phase out the use of antibiotics as a growth promotor in livestock. Moreover, in the same trend, the EU completely banned the use of antibiotics as growth promoters in livestock in 2006 [7]. Many others (United States, China, etc.) have implemented some guidelines to limit the use of antibiotics in animal science and production including shrimp farming [8,9]. Thus, the need for alternative solutions to support shrimp growth and health becomes a huge challenge. As an alternative to these restrictive measures, other alternatives are being explored to replace or at least reduce the quantity of antibiotics used in shrimp farming.

Essential oils (EOs) are complex mixtures of volatile chemicals released as secondary metabolites by aromatic plants or botanicals. From a chemical perspective, EOs consist of a combination of phenolics, terpenoids, terpenes and other bioactive (chemical) constituents [10]. Several EOs have demonstrated antimicrobial activity against fish and shrimp pathogenic bacteria [11,12,13,14,15,16,17]. Some studies consider individual in vitro screenings of botanical compounds against specific bacteria while others implement combined screenings of two or more substances. Interestingly, these substances are well recognized in exhibiting various mechanisms of action which might instigate their potential combination. Moreover, considering the chemical structure of each molecule and their functional groups, the antimicrobial activity of EOs cannot be explained by a unique mode of action but instead through a cascade of interactions in bacterial cells [18].

Phyto AquaBiotic (PAB) is a new blend of compounds derived from standardized EOs to support fish and shrimp health and growth. PAB contains three main molecules, namely thymol, carvacrol and cinnamaldehyde. These three active molecules hold an antimicrobial potential revealed by different studies against various Gram-negative bacteria from livestock, including fish and shrimp [19]. Since their single antibacterial activity is well known, it was valuable to assess the in vivo capacity of such a combination in reducing mortalities in the case of AHPND. Thus, the purpose of the present study is to evaluate the effect of PAB on the growth performance and survivability of *L. vannamei* after challenge against *V. parahaemolyticus*.

## 2. Materials and Methods

### 2.1. Diets and Tested Blend of EOs

For the feeding experiment, commercial pellets (Proconco brand) were used as the base diet. The commercial pellets had the following composition: 40% protein, 6% lipid and 4% ash. PAB, the tested additive in this study, is a blend of essential oils manufactured by Phytosynthese using liquid homogenizer equipped with an ultra turrax agitator. This product was incorporated into the basal diet at 0, 1 (PAB-1) and 2 (PAB-2) g/kg. To prepare the experimental diets, PAB was coated onto the commercial pellets, followed by the addition of 2% squid oil. This resulted in three experimental diets with different additive concentrations. The control diet consisted of commercial pellets coated with no PAB and only 2% squid oil. Diets were stored at 4 °C for the feeding experiment.

### 2.2. Shrimp and Experimental Conditions

The feeding experiment was conducted at the College of Aquaculture and Fisheries, Can Tho University, from November to December 2022. The shrimp used in the experiment had an initial weight of 0.9 ± 0.1 g per shrimp. The experiment was performed in outdoor Recirculating Aquaculture Systems (RAS) divided into three treatments with three replications. Each system consisted of four 1 m^3^ composite tanks filled with 0.8 m^3^ of water as well as a settling tank (0.5 m^3^) and a filter tank (0.5 m^3^) with substrate. The system was covered with a shade net while still being exposed to a 12 h light and 12 h dark photoperiod (12D:12L). During the experiment, average temperature ranged from 26.91 ± 0.02 °C to 26.95 ± 0.03 °C. Average pH values varied from 8.00 ± 0.01 to 8.05 ± 0.01. Salinity level was maintained at 15 ± 2 ppt. Alkalinity levels were kept above 100 mg CaCO_3_/L. Finally, total ammonium nitrogen ranged from 2.43 ± 0.36 to 2.92 ± 0.03 mg/L.

Shrimps were stocked at a density of 100 individuals per tank. They were fed four times a day, at 7–7:30 am, 10:30–11:00 am, 1:30–2:00 pm and 5:00–5:30 pm. The feeding amount was adjusted during the trial, ranging from 3% to 10% of the total body weight of the shrimp according to shrimp development stages. The feed amount was monitored by checking the bottom of the tanks for any excess feed remaining two hours after each feeding. This approach allowed for the minimization of overfeeding and ensured that the shrimp were fed close to satiation. The feed consumption in each tank was recorded daily by removing and weighing the excess feed (dry weight). However, the same feeding rate was considered for all treatments, so that the feeding rate did not affect the outcome of the experiment.

Throughout the 42-day feeding trial, any dead shrimp were removed daily, and the number of mortalities was recorded. At the end of the trial, all the shrimp in each tank were collected for further analysis or evaluation. Shrimp dying naturally during the stocking (only a few shrimp for a week or two weeks) of the feeding experiment resulted in biomass data.

### 2.3. Calculation of Growth Parameters

To assess the growth performance of the shrimp in the feeding experiment, the following formulas were used:

○ Daily weight gain (DWG, g/day) = (Wf − Wi)/42 days where Wi represents the initial weight of the shrimp (in grams) determined before the experiment and Wf represents the final weight of the shrimp (in grams) determined after 42 days;
○ Specific growth rate (SGR, %/day) = (Ln(Wf) − Ln(Wi)) * 100/42 days;
○ Feed conversion ratio (FCR) = consumed feed/weight gain where consumed feed is the total amount of feed consumed by the shrimp during the experiment and weight gain refers to the difference between the final weight and the initial weight of the shrimp;
○ Survival rate (SR, %) = (final number of shrimp/initial number of shrimp) * 100 where the final number of shrimp represents the count of surviving shrimp at the end of the feeding trial while the initial number of shrimp represents the count of shrimp stocked in each tank at the beginning of the experiment;
○ Biomass (kg m^−3^) = mean weight of shrimp x survival rate.

### 2.4. Challenge Experiment

#### 2.4.1. *Vibrio parahaemolyticus* Strain

The CM5 strain was used in this study. It was initially isolated from AHPND-infected shrimp in Ca Mau province and identified by PCR [20]. *V. parahaemolyticus* strain CM5 was recovered on nutrient agar plus 1.5% NaCl (NA, Himedia, Mumbai, India) for 16–24 h at 28 °C before being transferred to 10 mL of nutrient broth plus 1.5% NaCl (Himedia, Mumbai, India) for 24 h at 28 °C. The bacterial concentration was measured using a spectrophotometer (S-220, Boeco, Hamburg, Germany).

#### 2.4.2. Experimental Challenge

The challenge was adapted and conducted as described by [21]. On the 42nd day of the feeding experiment, 45 shrimp from two PAB treatments were challenged by immersion with *V. parahaemolyticus* at 1.2 × 10^7^ CFU/mL. Shrimp in the control treatment were used for positive treatment and were immersed in *V. parahaemolyticus* at 1.2 × 10^7^ CFU/mL. Shrimp in the control group were used for negative treatment and were immersed in sterilized nutrient broth plus 1.5% NaCl. Three replicates were performed for the challenge and negative treatments. After being challenged by *V. parahaemolyticus*, shrimp were transferred into a clean tank. The challenge experiment was conducted for 14 days. Shrimp were fed with experimental diets twice daily. The number of moribund shrimp was recorded every day. All moribund shrimp were streaked on TCBS agar for the isolation of *V. parahemolyticus*, and representative samples were tested for the presence of *V. parahaemolyticus* by PCR [20].

### 2.5. Total Vibrio Counts

The bacterial count was determined by the standard plate count method. The hepatopancreases (HP) were aseptically dissected. The HP were homogenized and serial 10-fold dilutions were performed with sterile saline solution (0.85% NaCl). A volume of 0.1 mL of the dilution was inoculated onto TCBS agar (Himedia, India) to enumerate the *Vibrio* spp. All of the TCBS plates were incubated at 28 °C for 24 h. Finally, all colonies of bacteria were counted and calculated as CFU/g unit.

### 2.6. PCR Method for Detection of V. parahaemolyticus

DNA was extracted from moribund shrimp HP samples. Extracted DNA was amplified by a two-tube nested PCR method that targets the tandem genes *PirA* and *PirB*. The chemical components and thermal cycling conditions were chosen according to the method of [13]. Electrophoresis results were recorded with a gel reader based on the 100 bp DNA ladder to determine the molecular weight; the *V. parahaemolyticus*-infected samples were expected to have a band of 230 bp.

### 2.7. Statistical Analysis

All data were presented as mean value ± standard deviation. Mean differences of parameters among treatments were tested by one-way ANOVA. The differences were considered significant at *p* < 0.05. In case of significant differences among treatments, Tukey’s post hoc test was performed for pairwise comparisons. Statistical analysis was conducted using MBI SPSS Statistics Version 21.

## 3. Results

### 3.1. Effects of PAB Supplement on Growth Performance of Whiteleg Shrimp

Growth performance and shrimp survival rate after 42 days of culture are presented in Table 1. The highest shrimp weight was recorded in the PAB-2 group (9.11 g/ind.) and was significantly higher than that of control treatments (8.19 g/ind.) (*p* < 0.05) but not statistically different compared to the PAB-1 group (8.7 g/ind.). Shrimp growth performance in the PAB-2 group (0.19 g/day) was also the highest while the lowest values were recorded in the control group (*p* < 0.05). FCR ranged from 1.08 to 1.15, but no significant difference in these values was observed among treatments (*p* > 0.05). The biomass obtained was 0.74–0.84 kg.m^−3^; PAB-1 and PAB 2 groups achieved significantly higher values compared to the control treatment. Growth parameters of experimental shrimp after 42 days of culture are presented in Table 1.

### 3.2. Effects of PAB Supplements on Disease Resistance of Whiteleg Shrimp

#### 3.2.1. Vibrio parahaemolyticus Challenge

##### Confirmation of Bacterial Infection

**Figure 1:**
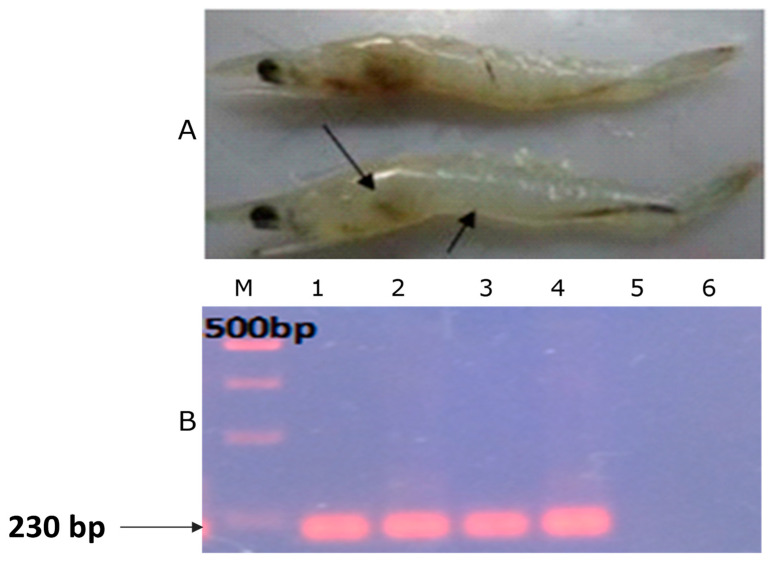
Confirmation of bacterial infection. (**A**) Clinical signs of experimental shrimp post-infection with *V. parahaemolyticus* with pale HP (double arrow) and empty gut (arrow); (**B**) PCR result of experimental shrimp post-infection with *Vibrio parahaemolyticus*. Lane M: DNA marker; lane 1: PCR result of positive control; lane 6: PCR result of negative control; lanes 2 (PAB-1 treatment), 3 (PAB-2 treatment), 4 (positive treatment) and 5 (negative treatment): shrimp DNA isolated from different treatments of the challenge experiment.

Clinical signs of whiteleg shrimp challenged with *V. parahaemolyticus* were recorded as pale-to-white HP, soft shells and empty guts (Figure 1A). The challenged shrimps were also collected and detected for *V. parahaemolyticus* by PCR (Figure 1B). Figure 1B shows that challenged shrimps presented a bright positive band (230 bp) for *V. parahaemolyticus*. Since negative control shrimp (lane 5) were not experimentally infected with *V. parahaemolyticus*, the band did not appear.

##### Cumulative Mortality of Shrimps Challenged with *V. parahaemolyticus*

**Figure 2:**
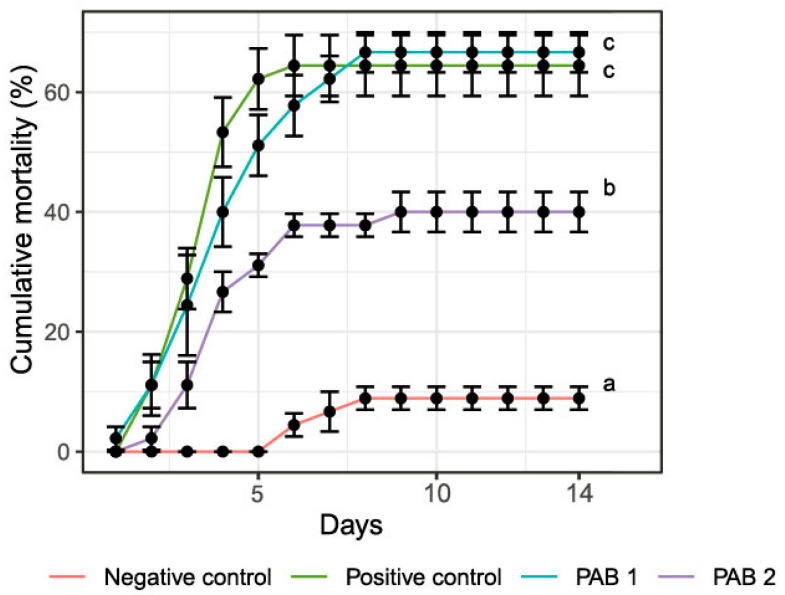
Cumulative mortality of experimental shrimp challenged with *V. parahaemolyticus* on day 42 of feeding experiment.

During the 14-day challenge experiment, the cumulative mortality of shrimp fed with PAB-2 (40%) was significantly lower than the cumulative mortality of the positive control group, which was 66.7% (*p* < 0.05) (Figure 2). On the other hand, for the PAB-1 treatment, the cumulative mortality was measured at 64.4%, which was slightly lower than the cumulative mortality of the positive treatment (66.7%).

#### 3.2.2. Bacterial Density from Shrimp Hepatopancreas

Total *Vibrio* densities in the HP of experimental shrimp is shown in Table 2. On the sampling day, the highest total *Vibrio* counts were observed in the positive treatment. Although the *Vibrio* count was lower in all supplement treatments (*p* < 0.05), there was no statistical difference in *Vibrio* densities among supplement treatments.

## 4. Discussion

Presently, the use of plant-based additives in aquafeed represents a fundamental approach towards promoting sustainability in aquaculture [18]. Many studies have explored the use of these botanicals in aquaculture in different forms (raw herbs/plants, dry extract, EOs, etc.), singularly or in combination, revealing interesting bio-properties and benefits in fish and shrimps [11,22,23]. In this study, a mixture of compounds from EOs were used to evaluate the growth performance of shrimps and their effect on resistance against AHPND was determined. Evidence of using EOs in aquaculture is demonstrated in the literature and highlights growth performance in fish and shrimps. For instance, dietary carvacrol and thymol significantly enhanced growth performance (weight gain and FCR) in channel catfish (*Ictalurus punctatus*) [13]. In another study on rainbow trout, dietary thymol at different dosages (1.5 and 2.5 g/kg) improved growth performance as reflected by the final weight, weight gain and specific growth rate [14]. In the same species, Ref. [15] demonstrated the effect of thymol (6 g/kg) and carvacrol (12 g/kg) on the significant improvement in growth performance parameters, mainly FCR. In Nile tilapia, cinnamaldehyde and thymol were also fed at different dosages and showed an increase in growth parameters such as body weight gain, average daily weight gain, total feed intake and specific growth rate [16]. Moreover, in shrimps, EOs were also tested to evaluate beneficial properties such as growth and feed efficiency performance [24,25]. A study performed on *L. vannamei* revealed that thyme EOs significantly improved growth performance at a dose of 1% [25]. In contrast, another study demonstrated that oregano EOs showed no effect on this shrimp species [24]. In addition, trans-cinnamaldehyde significantly (*p* < 0.05) improved growth performance by enhancing the activity of digestive enzymes in shrimp [26]. Of course, the effect of plant-based additives in biological organisms (fish, shrimps, etc.) depends on different factors such as composition, animal stages, dosages, etc. [27]. In this study, PAB, which is an EO combination with three main molecules (thymol, carvacrol and cinnamaldehyde), exhibited an improvement in growth performance at two different dosages (0.1% and 0.2%). Over 42 days of feeding to *L. vannamei*, it was demonstrated that PAB at 2 kg/t was able to significantly increase final weight, daily weight gain and total biomass (*p* < 0.05). A feed additive is all the more attractive if, in addition to other specific properties (antioxidant, antimicrobial, etc.), it improves growth performance. In this case, notwithstanding the potential antimicrobial effect of such a product, it demonstrated an increase in various factors of growth measurement. Moreover, to achieve growth enhancement, it is common to see the incorporation of high dosages of botanicals or additives in aquaculture (above 0.2%) [15]. In this study, a low dosage (0.1%) also improved growth performance compared to the unsupplemented group. This finding is quite appealing in aquafeed in ensuring feed cost and return on investment (ROI) optimization. Since EOs and mixtures are combinations of various aromatic flavoring molecules, this improved growth performance achieved at such a low dosage could be linked to their strong appetizing nature and feed intake increase potential [28,29]. In addition, bioactive compounds of plants induce the secretion of digestive enzymes which increase feed consumption and absorption of nutrients [29,30].

Active molecules from various aromatic plants or EOs are known to exhibit a range of antimicrobial activities [30,31]. Many active molecules, including those that formulate PAB such as cinnamaldehyde, carvacrol and thymol, are widely listed as well as antibacterial compounds [30,31,32]. In this study, PAB at the dosage of 2 g/kg significantly reduced mortalities (40% vs. 66.7%) compared to the positive control when challenged against *V. parahaemolyticus* (*p* < 0.05). Several studies revealed, in vitro, the antibacterial properties of such active molecules (mainly cinnamaldehyde) throughout a range of diverse Gram-positive and Gram-negative pathogenic bacteria including *Vibrio* sp. [32,33,34,35,36]. Moreover, many in vivo studies were also performed individually or in combination with different active molecules derived from EOs. From these studies, including the present study, relevant results are shown related to a reduction in mortalities in fish and shrimps [13,14,17,29,37]. For instance, a blend of such molecules (thyme and cinnamon) was shown to significantly reduce mortality in *L. vannamei* when challenged against *V. parahaemolyticus* [17]. This finding is consistent with our results on the same species of shrimp, regardless of initial shrimp size, trial duration or pathogenic bacterial load. In another species in which a challenge against *E. ictaluri* was performed, a significantly higher survival was observed in catfish that received trans-cinnamaldehyde at the levels of 15 and 20 mg/kg compared to a control group (49.12% and 65.52% survival vs. 11.11% survival) [37]. In a study performed on scallops, cinnamaldehyde had a significant protective effect against *V. anguillarum*, confirmed by increased survival [38]. Moreover, in a striped snakehead (*Channa striatus*) challenged against *Aphanomyces invadans,* the percentage of mortality was significantly lower in a dose-dependent manner (5–15%) compared to an unsupplemented group after being fed cinnamaldehyde at rates of 5, 10 and 15 mg/kg [39]. Protected cinnamaldehyde under lipid bilayers of liposome also significantly enhanced the survival rates of *Streptococcus agalactiae*-, *Aeromonas hydrophila*- and *V. vulnificus*-infected zebrafish [40]. The same applies to [27], in which a reduction in mortalities of a group of Nile tilapia (*Oreochromis niloticus*) challenged against *Streptococcus agalactiae* was demonstrated. Thymol and carvacrol are also potent molecules in this product, known for their beneficial antibacterial activities. Chemically, both thymol and carvacrol share the same chemical formula (C_10_H_14_O) with the hydroxyl group in thymol located in the meta position whereas, in carvacrol, it is situated in the ortho position [41]. Evidence has been demonstrated in many species of fish and shrimp as single molecules or in combination [42]. For example, in trout fish challenged against a virulent strain of *Aeromonas hydrophila*, *Thymus vulgaris* EOs at 2 mL/kg of feed significantly increased the survival rate (31.58%) compared to the control which was challenged and not fed thymol (4.76%) [43]. The inclusion of oregano EOs (including thymol and carvacrol; the concentration was not revealed) at 5 to 20 g/kg of diet resulted in a significant improvement in the 10-day cumulative survivability of fish challenged against *A. hydrophila* (*p* < 0.05) in all oregano EO-supplemented fish groups (37–57.5%) with respect to a control group (0%) [44]. In another study, tilapia supplemented with 1% of thyme powder (from a local market) showed a significant enhancement in survival rate (78% vs. 39%) after a challenge with *S. iniae* [45]. Mortality due to infection by *A. hydrophila* was also reduced by 40% in African catfish (*Clarias gariepinus*) fed 1% of thyme powder compared to a control. Moreover, [29] demonstrated the potential of feeding oregano (*Origanum onites L*.) EOs (92.59% of carvacrol) in doses of 0.125 to 3 mL/kg to significantly reduce mortality in *O. mykiss* after being challenged against *Lactococcus garvieae.* In shrimps, an assessment of micro-encapsulated thymol EO (1%) was carried out by [45] to mitigate the negative effects of White Spot Diseases (WSD). It was demonstrated that this EO revealed the absence of clinical signs of White Spot Syndrome Virus (WSSV) infection and increased the survivability of the supplemented group compared to unsupplemented ones.

However, the mechanisms by which these active molecules derived from EOs exhibited their antimicrobial activity were not totally elucidated. It is thought that EOs may exert a structural influence on the bacterial membrane and its transport system [34,46]. One of the main mentioned modes of action of EOs is membrane compromission leading to a disrupted osmotic pressure and intracellular leakage with eventual cell destruction [34,47]. In addition, another proposed mechanism of action for EOs is their capability to inhibit the bacterial efflux system. The bacterial efflux system consists of specialized channel proteins situated on the bacterial membrane. These proteins play a vital role in eliminating harmful compounds, including antibiotics, from the intracellular environment [48,49]. Thymol, carvacrol and cinnamaldehyde were individually demonstrated to exhibit such antimicrobial properties through the above-cited mechanisms. For instance, Ref. [50] proposed that the mode of action of thymol as an antimicrobial mediator could be mostly ascribed to destructive impacts on the generation of adenosine triphosphate (ATP) and the cellular cytoplasmic membrane. Moreover, it interacts with the cell membrane by hydrogen bonding, rendering the membranes and mitochondria more permeable and disintegrating the outer cell membrane. For cinnamaldehyde, in addition to previously identified modes of action, it has the capacity to inhibit the biofilm formation of different bacterial strains [32,41]. Cinnamaldehyde has also been studied for its potential role in quorum quenching, a mechanism that aids in disrupting bacterial communication systems [32,51]. Ref. [51] demonstrated, in a *V. harveyi* model, that cinnamaldehyde and cinnamaldehyde derivatives interfere with AI-2-based quorum quenching by decreasing the DNA-binding ability of LuxR. Thus, the combined modes of action of these main active molecules from EOs can differently interfere with the growth and pathogenicity of bacteria such as *V. parahaemolyticus*, leading to low mortality compared to control.

Mixtures of plant-based additives may have an impact on pathogenic/opportunistic bacteria in the digestive tract of fish or shrimps as components of nutraceutical diets [52]. In this study, the *Vibrio* load was numerically lower in the HP of the group fed with PAB in comparison to the group not fed with PAB. Considering the bacteriostatic activity of all compounds formulating PAB [19], the reduction in pathogenic bacteria strain growth and load might constitute a potential hypothesis of this evidence. In addition, bacteria of the *Vibrio* genus are considered opportunistic bacteria found in shrimp organs such as the intestine and HP. In the digestive tract of healthy *L. vannamei*, the abundance of such bacteria is generally either low or maintained at a specific level that allows for a balanced coexistence with other microbial species in the shrimp’s gut [53]. Under various conditions, such as challenge, malnutrition or stress, the abundance of beneficial bacteria in the shrimp’s digestive tract can decrease, leading to a vacant ecological niche, which opportunistic or pathogenic bacteria can exploit to survive and proliferate. Therefore, disease outbreaks may occur with subsequent mortalities [54,55]. Indeed, carefully monitoring the abundance of these bacteria in shrimp culture is crucial in preventing potential epidemics. This reduction was already observed in different fish and shrimp species targeting various bacterial strains and organs [56]. For instance, the administration of cinnamaldehyde to Pacific white shrimp can reduce the number of *Vibrio* bacteria both in the intestine and HP [56]. In shrimp culture, the majority of *Vibrio* bacteria with green colonies consist of opportunistic pathogens capable of causing vibriosis disease. These pathogens tend to accumulate in the HP and intestine of shrimps. In the current study, the load of *Vibrio* spp. was assessed to ascertain the presence of this group of strains. The same trend of reduction in the presence of PAB was observed. Ref. [37] assessed the effect of cinnamaldehyde on *V. anguillarum* clearance in the hemolymph. Findings showed that cinnamaldehyde significantly enhanced *V. anguillarum* clearance compared to the control pre- and post-treatment. In another study, feeding a micro-encapsulated blend of EO and organic acids reduced the abundance of *Aeromonas hydrophila* and *Streptococcus* sp., which are opportunistic pathogenic bacteria in the gut of rainbow trout [57]. The effect of powdered cinnamon bark (from a local market) on antibacterial capacity in the intestines of European sea bass was determined [58]. It was demonstrated that, in fish treated with powdered cinnamon bark, *Vibrio* spp. count was lower compared to fish not supplemented with any cinnamon powder (*p* < 0.05). Although, in this study, this significance was not seen, the trend remains the same and highlights the efficiency of active molecules, mainly in reducing opportunistic pathogens such as *Vibrio parahaemolyticus*.

Finally, other important parameters that should be carefully considered are the optimal dosage of PAB necessary to obtain the most significant effect on many parameters. It is important to note that, in aquaculture, species are numerous with huge biological variation in growth (stages), and conditions of culture are not always the same. However, it is obvious that PAB exhibited a potent antibacterial effect against *Vibrio parahaemolyticus* in shrimps.

## 5. Conclusions

PAB, a mixture of active molecules from EOs, exhibited beneficial effects on *L. vannamei* at different dosages. In this study, a 2 g/kg dosage of PAB significantly enhanced growth performance parameters such as final weight, daily weight gain and total biomass and numerically enhanced all other parameters (FCR, SGR and Vibrio count in HP). Additionally, a significant reduction in mortalities due to *V. parahaemolyticus* challenge was observed in comparison to the challenged control group (positive control). A numerically lower Vibrio count was also demonstrated in infected shrimps fed PAB compared to the infected group without supplementation. Based on these findings, the bacteriostatic effect of PAB could probably be pointed out through a potential increase in bacterial membrane permeability and disruption of cell membrane integrity. Overall, this study revealed that PAB inclusion at 2 g/kg could significantly improve growth performance and reduced mortality which might occur during AHPND outbreaks.

## Figures and Tables

**Table 1:** Growth parameters of whiteleg shrimps over 42 days.

Parameters	Treatments		
	Control	PAB-1	PAB-2
Wi (g/ind.)	1.02 ± 0.01	1.02 ± 0.01	1.04 ± 0.04
Wf (g)	8.19 ± 0.45 ^a^	8.70 ± 0.25 ^ab^	9.11 ± 0.12 ^b^
DWG (g·d^−1^)	0.17 ± 0.01 ^a^	0.18 ± 0.01 ^ab^	0.19 ± 0.00 ^b^
SGR (%·d^−1^)	4.98 ± 0.19	5.00 ± 0.09	5.21 ± 0.13
FCR	1.15 ± 0.10	1.14 ± 0.01	1.08 ± 0.10
Biomass (kg·m^−3^)	0.76 ± 0.04 ^a^	0.81 ± 0.01 ^b^	0.80 ± 0.01 ^b^

(Wi: initial mean weight, Wf: final mean weight, DWG: mean daily weight gain, SGR: specific growth rate, FCR: feed conversion ratio, d: day). Values are means of three replicates ± SD. Within a row, values with the same letters are not significantly different (*p* > 0.05).

**Table 2:** Total *Vibrio* count in shrimp HP (×10^6^ CFU/g).

Groups	*Vibrio* Count (×10^6^ CFU/g)
Negative control	4.49 ± 3.43
Positive control	8.06 ± 4.69
PAB-1	5.77 ± 2.44
PAB-2	5.28 ± 3.89

Values are means of nine replicates ± SD.

## Data Availability

Data availability statements are available under request via the above email.
